# Phytonutrient intakes in relation to European fruit and vegetable consumption patterns observed in different food surveys

**DOI:** 10.1017/S0007114514001950

**Published:** 2014-08-11

**Authors:** David R. Tennant, Julia Davidson, Andrea J. Day

**Affiliations:** 1 Food Chemical Risk Analysis, 14 St Mary's Square, BrightonBN2 1FZ, UK; 2 Amway (UK) Limited, St Anne's House, Caldecotte Lake Drive, Caldecotte Business Park, Caldecotte, Milton KeynesMK7 8JU, UK; 3 School of Food Science and Nutrition, University of Leeds, LeedsLS2 9JT, UK

**Keywords:** Fruit and vegetable intakes, Flavonoids, Carotenoids, National Diet and Nutrition Survey

## Abstract

Fruit and vegetables make an important contribution to health, partly due to the composition of phytonutrients, such as carotenoids and polyphenols. The aim of the present study was to quantify the intake of fruit and vegetables across different European countries using food consumption data of increasing complexity: food balance sheets (FBS); the European Food Safety Authority (EFSA) Comprehensive Database; individual food consumption data from the UK National Diet and Nutrition Survey (NDNS). Across Europe, the average consumption of fruit and vegetables ranged from 192 to 824 g/d (FBS data). Based on EFSA data, nine out of fourteen countries consumed < 400 g/d (recommended by the WHO), although even in the highest-consuming countries such as Spain, 36 % did not reach the target intake. In the UK, the average consumption of fruit and vegetables was 310 g/d (NDNS data). Generally, phytonutrient intake increased in accordance with fruit and vegetable intake across all European countries with the exception of lycopene (from tomatoes), which appeared to be higher in some countries that consumed less fruit and vegetables. There were little differences in the average intake of flavanols, flavonols and lycopene in those who did or did not meet the 400 g/d recommendation in the UK. However, average intakes of carotenoid, flavanone, anthocyanidin and ellagic acid were higher in those who consumed >400 g/d of fruit and vegetables compared with those who did not. Overall, intakes of phytonutrients are highly variable, suggesting that while some individuals obtain healthful amounts, there may be others who do not gain all the potential benefits associated with phytonutrients in the diet.

WHO recommend the consumption of at least 400 g of fruit and vegetables a day, and many European adults are aware of the communication of this advice^(^ ^1^ ^)^. An emerging science indicates that habitual low consumption of fruit and vegetables may be considered a risk factor for chronic diseases, such as CVD; indeed, the WHO estimates that low intake is responsible for approximately 11 % of IHD deaths and 9 % of stroke deaths^(^ ^2^ ^)^. Plant-based foods provide an advantage to health, when increasing proportions are incorporated into the diet, not only due to the replacement of energy-dense foods and increases in fibre and micronutrient content, but also due to the diversity of phytochemicals that are consumed.

A plethora of phytochemicals are produced by plants; these secondary metabolites are used to protect the plant from UV light, as insect or pest defence, and to produce colour and other organoleptic characteristics^(^ ^3^ ^)^. They are not classically regarded as nutrients (as they are not required for essential growth or maintenance of life); however, their sheer abundance in plant-based foods and significant potential to improve health mean they are regarded as phytonutrients. Many different, and chemically unrelated, phytonutrients exist in plant-based foods, including carotenoids, polyphenols, indoles, glucosinolates, organosulfides, betalains, phytosterols and others. All of these have been studied for their potential bioactivity in relation to health, initially due to interest in antioxidant properties of many of these compounds.

Carotenoids are found in coloured vegetables and fruit, and are often responsible for the red, orange and yellow colours in these foods. Over 600 carotenoids have been identified, but only twenty are found in human plasma and tissue, and these are from five classes: lycopene; α-carotene and β-carotene; β-cryptoxanthin, lutein and zeaxanthin. Lycopene is predominantly found in tomatoes and tomato-based products, while the others are found in varying quantities across leafy green vegetables, orange-fleshed vegetables and fruit, and maize products. Lutein and zeaxanthin can also be found in egg yolk derived from the chicken's feed. β-Cryptoxanthin, α- and β-carotene all have provitamin A activity, whereas lycopene, lutein and zeaxanthin do not. Promising areas of research on bioactivities of the carotenoids relate to eye and skin health, limiting DNA damage and cardioprotection^(^ ^4^ ^–^ ^6^ ^)^.

Polyphenols, characterised by one or more phenolic rings, are divided into flavonoids, hydroxycinnamates, hydroxy-benzoates, stilbenes and lignans. Over 9000 flavonoids alone have been identified, and this grouping can be further subdivided into six main classes, which include flavonols, flavones, flavanones, flavanols, isoflavones and anthocyanidins. Flavonols and flavanols occur ubiquitously across plant-based foods; herbs in the diet mainly provide the flavones consumed; flavanones are mainly found in citrus foods; isoflavones are predominantly from soya products; and anthocyanidins are the brightly coloured reds and blues, particularly in berries. Hydroxycinnamates and hydroxy-benzoates are also found ubiquitously across fruit and vegetables, but there is increasing interest in the more complex hydroxy-benzoic acid derivatives, ellagitannins and ellagic acid^(^ ^7^ ^)^. Ellagitannins are referred to as hydrolysable tannins as on acid hydrolysis, and after bacterial metabolism in the intestinal tract, they are broken down to ellagic acid. Ellagitannins and ellagic acid are found in berries, pomegranate, nuts (walnuts and pecans) and oak-aged alcoholic beverages (e.g. red wines and cognac). Recent research focus for many of the polyphenols is on cell signalling pathways, inhibition of oxidative enzymes, increasing endothelial NO synthase activity, and decreasing adhesion molecules and platelet aggregation^(^ ^8^ ^–^ ^10^ ^)^.

A recent study in the USA has found that intakes of phytonutrients (carotenoids, flavonoids and ellagic acid) were significantly lower among adult individuals who consumed less than the daily amounts of fruit and vegetables recommended in the dietary guidelines of the US Department of Health and Human Services^(^ ^11^ ^)^. Food-based dietary guidelines have been established for many regions and countries across the world and are collated by the FAO^(^ ^12^ ^)^. In Europe, although some individual countries may be encouraged to consume more than six portions of fruit and vegetables per d^(^ ^13^ ^)^, the European Union supports the WHO recommendation for at least 400 g/d. Fruit and vegetable consumption patterns may be expected to vary widely between global geographical regions depending on food availability and culture. As a consequence, the degree to which national phytonutrient intakes relate to compliance with local dietary guideline(s) can be expected to vary. The purpose of the present study was to determine population intake of specific phytonutrients in relation to fruit and vegetable consumption across Europe. Detailed food consumption data are not available for all European countries and so a hierarchical approach was adopted that used simple food balance sheet (FBS) data for all European countries and summarised individual data for countries represented in the European Food Safety Authority database. Finally, using more detailed (4 d diary) dietary data, collected from individuals as part of the UK National Diet and Nutrition Survey (NDNS)^(^ ^14^ ^)^, we determined the intake of phytonutrients related to fruit and vegetable consumption in the UK.

## Methods

### Approach

The availability of data on food consumption is extremely variable between countries, from individual weighed diary records at one extreme down to simple population statistics at the other. This means that the level of detail that can be applied to analysing differences in phytonutrient intakes must be adapted according to the data available. The present study used a three-tiered structured approach:

1 Tier 1 examines the differences in the consumption of fruit and vegetables between countries at a simple per capita level. This allows international comparisons on a population basis, but does not permit assessment at the individual level and, in particular, consideration of high- or low-level consumers of particular foods or groups of foods. National food consumption patterns can then be compared with international and national dietary guidance values.
2 Tier 2 makes use of more detailed summary statistics that provide distributional data (means, percentage consuming, and upper and lower percentiles) for food categories and individual foods. These data make it possible to identify high- and low-level consumption of particular foods or groups of foods, and to draw conclusions about the range of potential intakes of phytonutrients in relation to international and national dietary guidance values.
3 Tier 3 utilises individual food consumption data that are available for certain national populations. It is then possible to estimate each individual's intake of specific phytonutrients and to consider those meeting national or international guidelines separately from those who do not. Sub-population groups can also be considered, such as different age groups, sex, or high or low body weights.

### Phytonutrient levels

The UK Food Standards Agency Nutrient Databank^(^ ^15^ ^)^ was used to determine the carotenoid content of foods; in addition, a limited amount of data was extracted from the US Department of Agriculture National Nutrient Database^(^ ^16^ ^)^. Data for flavonoids and ellagic acid were determined, using chromatographic data only, through advanced searches from the Phenol-Explorer database (version 3)^(^ ^17^ ^)^. Phenol-Explorer provides data both as individual glycosylated compounds and/or as the parent aglycone after hydrolysis. The glycosylated values were predominantly used, which were converted to the aglycone equivalent for individual flavonoid classes. The hydrolysed value was used for ellagic acid, which is particularly important as hydrolysis allows the identification of content from larger hydrolysable tannins present in the food sample. Any food item missing from Phenol-Explorer was assessed from the US Department of Agriculture Database^(^ ^16^ ^)^.

Tea mainly refers to black or green tea infusions; although black tea dominates the market in the UK, this is not the case across different European countries. Detailed information about the relative proportions consumed in different countries was not available, and so we used a simple model of 50 % of each tea type. This assumption does not significantly affect flavonol intake, and would only marginally overestimate flavanol monomer intake in high black tea-consuming countries. Polymeric flavanols (theaflavins and thearubigins) were not quantified in the present study, and are the dominant flavonoid derivative in black tea. For similar reasons, wine intake across Europe was assumed to be 50 % of red wine; however, consumption of a greater proportion of red wine in these case populations will cause an underestimation of intake of the associated flavonoids.

All of the information was compiled into one database of phytonutrient levels in the principal categories of foods (see online supplementary Table S1). Care was taken to ensure that foods containing the highest phytonutrient concentrations were identified together with foods that contain lower levels that might contribute more to the intake because of larger amounts consumed.

### Food consumption data

Tier 1 data were taken from the FAO's FBS that are available for primary food products for many countries and covering all continents^(^ ^18^ ^)^. Definitions of ‘fruit’ and ‘vegetables’ were based on WHO/FAO guidelines^(^ ^2^ ^)^ that excluded cereals, potatoes, beverages, etc. The data included many specific food items such as certain fruit and vegetables, for example, ‘tomatoes’ and ‘root vegetables’, as well as broad food categories such as ‘other vegetables’. However, only average per capita supply (production+imports − exports − wastage − non-food use) consumption data are provided (kg/person per annum).

Tier 2 data were taken from the European Food Safety Authority (EFSA) ‘Comprehensive Food Consumption Database’ for adults^(^ ^19^ ^)^. The database covers up to five age groups in twenty-two different European Union member states. The Comprehensive Database includes twenty broad food categories that are subdivided into more specific food types. The data are provided as consumption for the entire population or for consumers only and also expressed as per kg body weight. For each food category, the database provides the number of consumers in the survey (and percentage consuming), mean and standard deviation, and median and 5th, 10th, 95th, 97·5th and 99th percentile consumption. Only five of the highest-level FoodEx codes used to describe the Comprehensive Food Consumption data relate to fruit and vegetables. Potatoes, like cereals, are not included as fruit and vegetables for health target purposes, but have been included in the calculations of phytonutrient intakes. Fruit juices are included in health guidance but only up to 150 ml, and beans and pulses count as up to one portion (80 g)^(^ ^20^ ^)^. To calculate total fruit and vegetable consumption in each European Union country, the contributions from vegetables and vegetable products; legumes, nuts and oilseeds (capped at 80 g); fruit and fruit products, and fruit and vegetable juices (capped at 150 g) had to be combined. They could not be simply added as this would distort total consumption at the upper percentiles. Instead, @RISK software^(^ ^21^ ^)^ was used to combine the distributional data (ranges and percentiles) for adults in a probabilistic model. Population statistics were drawn from the resulting distribution of total fruit and vegetable consumption. For the estimates of intake of phytonutrients, food consumption data at the more detailed FoodEx level L2 were applied (see online supplementary Table S2). Wine, but not tea, was taken into account for determining phytonutrient intake.

Tier 3 data were national individual-based food consumption survey data for adults aged over 18 years (*n* 1016) from the UK NDNS conducted in 2008–2009^(^ ^14^ ^)^. The data are collected by individuals who recorded their (or a dependant's) daily food consumption on an item-by-item basis over 4 d (with all days being equally represented across the 2 years). Using such survey data, it is possible to allocate a phytonutrient level to each food item and so estimate the total intake of that individual over a given period of time. It is then possible to derive population statistics for the entire population or for selected subgroups. To maintain consistency with healthy eating guidelines, consumption of fruit juices qualifying for inclusion as fruit and vegetables was limited to 150 ml and consumption of pulses limited to one portion per d (80 g). Consumption data of all other fruit and vegetables not included in WHO guidelines, such as potatoes, teas and wine, were excluded from the estimates of fruit and vegetable consumption. However, these limitations were not taken into consideration when estimating intakes of phytonutrients.

### Statistical analysis

Average fruit and vegetable consumption and phytonutrient intakes were compared using Student's *t* test (two-sample assuming equal variances), taken from the Microsoft Excel data analysis add-in. The *P*(*T*≤ *t*) two-tail statistic, giving the probability that a value of the *t* statistic would be observed that is larger in absolute value than *t*, was considered significant if < 0·05. Therefore, the null hypothesis that the means were equal was rejected if the value of *P* was < 0·05.

## Results

**Fig. 1:**
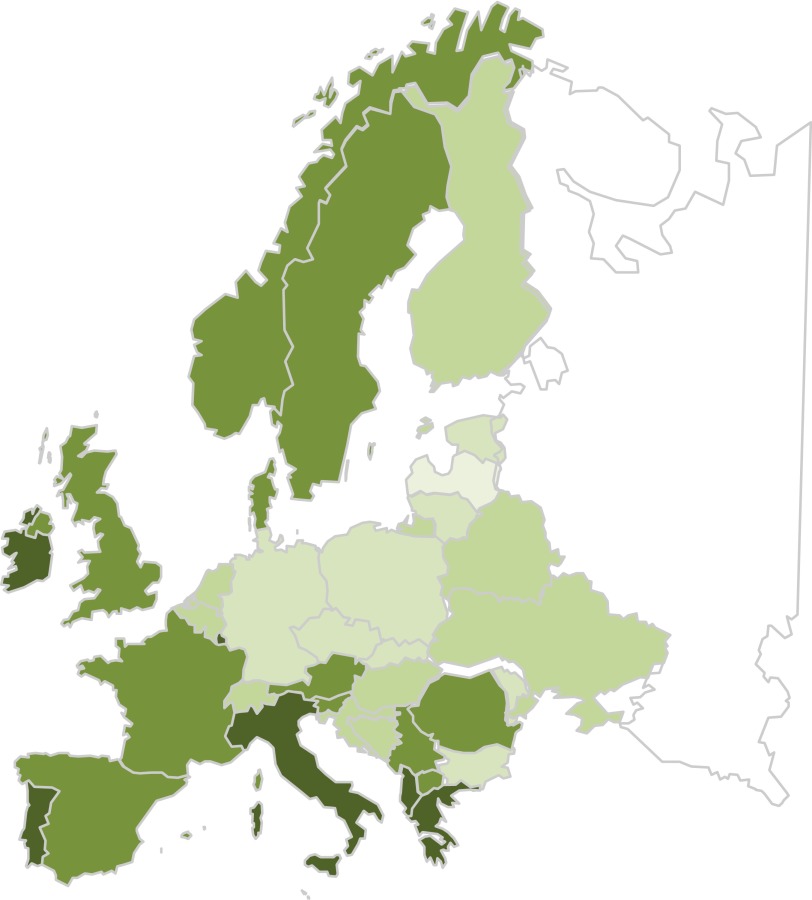
Population average consumption of fruit and vegetables in European countries, based on the food balance sheet data (g/d)^(^ ^18^ ^)^. 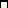, 100–200; 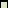, 200–300; 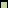, 300–400; 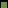, 400–500; 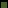, 500+. A colour version of this figure can be found online at http://www.journals.cambridge.org/bjn

At tier 1, using the FBS data, total fruit and vegetable consumption was estimated for forty-five countries across eastern, central and western Europe (Fig. 1). The average amount of fruit and vegetables consumed ranged from 192 g/d in Latvia to 824 g/d in Greece. The overall average consumption was 429 g/d, which exceeds the WHO Countrywide Integrated Non-communicable Diseases Intervention Dietary Guide recommendation of 400 g/d^(^ ^22^ ^)^. Of the forty-five countries, twenty-five had fruit and vegetable consumption equal to or greater than 400 g/d and twenty had less than that amount. Fruit and vegetable consumption was highest in southern Europe (600 g/d) followed by northern Europe (434 g/d) and western Europe (387 g/d), and lowest in eastern Europe (310 g/d). Total fruit and vegetable consumption data excluded starchy foods such as potatoes and pulses, which may be valuable sources of phytonutrients. The food consumption data did take some processing, but not wastage (e.g. vegetable peeling), into account in the home and so tended to overestimate true consumption. Products processed and then re-exported in other foods, such as the manufacture of juice-based drinks, were not identified.

**Fig. 2:**
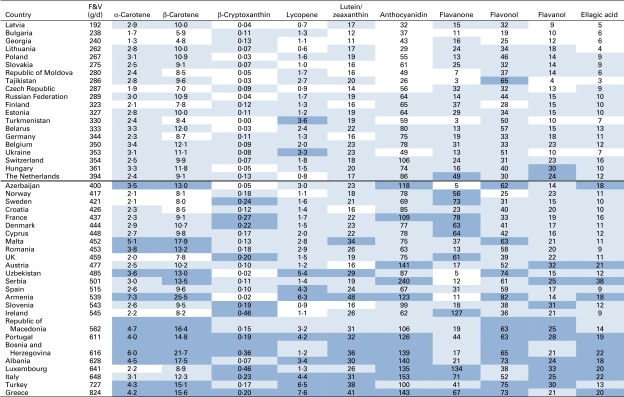
Phytonutrient intakes in European countries in relation to fruit and vegetable (F&V) consumption from the WHO's food balance sheets. F&V consumption taken from the food balance sheet data^(^ ^18^ ^)^. Phytonutrient intake (mg/d) is the product of F&V consumption and phytonutrient levels from online supplementary Table S1. The values above the double line represent the countries that consume an average amount of < 400 g of F&V. Dark, light and no shading indicate upper 25th, middle 50th and lower 25th percentiles, respectively, for consumption of each phytonutrient. A colour version of this figure can be found online at http://www.journals.cambridge.org/bjn

Average intakes of phytonutrients were calculated for each country and arranged according to the level of fruit and vegetable consumption (Fig. 2). Upper and lower 25th percentiles were identified with a shaded-colour code. Countries with higher consumption of fruit and vegetables generally had significantly higher intakes of phytonutrients. The broad food categories assigned in the FBS system will tend to overestimate phytonutrient intakes, but the differences between countries and trends will probably remain valid. There was a positive association between β-carotene and all the other carotenoids, except lycopene, which would be expected due to their common occurrence in similar fruit and vegetables. There was no strong association between any of the polyphenols assessed, with the exception of ellagic acid and anthocyanins, which have very similar food sources.

**Fig. 3:**
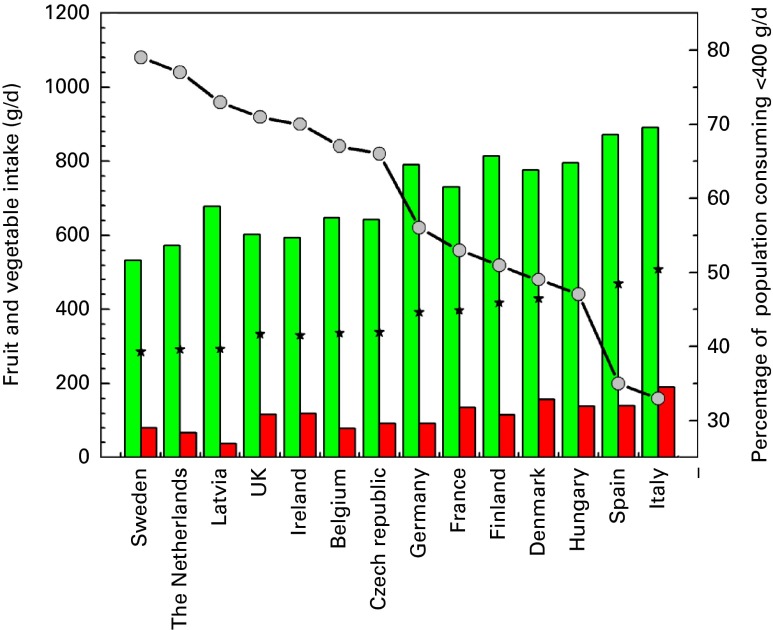
Fruit and vegetable consumption in European Union countries based on the European Food Safety Authority Comprehensive data^(^ ^19^ ^)^. Primary *y*-axis: values are means (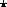), with bars representing 5 % (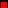) to 95 % (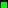) intake. Secondary *y*-axis: 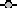, percentage of the population consuming < 400 g/d of fruit and vegetables. A colour version of this figure can be found online at http://www.journals.cambridge.org/bjn

At tier 2, using the EFSA data, European countries were ranked according to the degree to which the international guidelines for fruit and vegetable consumption were met by consumers (Fig. 3). Consumption of fruit and vegetables ranged from 0 to nearly 1400 g/d. However, extremes of consumption probably reflect the short-term duration of surveys and are probably not sustained in the longer term. The average consumption was 375 g/d, with nine out of fourteen countries consuming on average less than the recommended amount. The percentage of the population who consumed < 400 g/d ranged from < 36 % in Spain and Italy to >70 % in Ireland, Latvia, The Netherlands, Sweden and the UK.

**Fig. 4:**
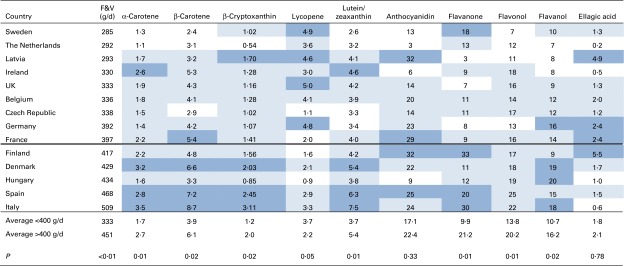
Phytonutrient intakes in European Union countries in relation to fruit and vegetable (F&V) consumption from the European Food Safety Authority (EFSA) Comprehensive Food Consumption Database. F&V consumption taken from the EFSA data^^(^^19^^)^^. Phytonutrient intake (mg/d) is the product of F&V consumption and phytonutrient levels from online supplementary Table S1. The values above the double line represent the countries that consume an average amount of < 400 g of F&V. Dark, light and no shading indicate upper 25th, middle 50th and lower 25th percentiles, respectively, for consumption of each phytonutrient. A colour version of this figure can be found online at http://www.journals.cambridge.org/bjn

Intakes of phytonutrients were estimated by multiplying the phytonutrient levels by the average consumption of the detailed food categories at FoodEx level L2 (see online supplementary Table S2). The results were ranked by total fruit and vegetable consumption and subdivided by countries with average fruit and vegetable consumption above or below the recommended levels (Fig. 4). The upper and lower 25th percentiles of phytonutrients were identified with a shaded-colour code. With the exception of lycopene, the intake of phytonutrients was on average 1·5 times higher (range 1·1–2·1) in countries with higher consumption of fruit and vegetables. These differences in national averages between high and low fruit and vegetable-consuming countries were statistically significant (*P*< 0·05), except for anthocyanins and ellagic acid. Conversely, lycopene was significantly higher (1·7 times) in low fruit and vegetable-consuming countries. Similar to the FBS data, there was an association between all carotenoids, except lycopene, but little relationship between the polyphenols.

Tier 3, using the NDNS data, modelled established fruit and vegetable consumption for each adult in the UK, and determined whether or not each individual met the national and international dietary guidelines. The details of the consumption of fruit and vegetables by 1007 adults in the 2008/2009 UK NDNS are provided in Table 1. Total consumption ranged from < 1 to 1400 g/d and averaged at 310 g/d. A small number of individuals reported no consumption of fruit and vegetables, which may be related to survey duration, survey design or misreporting. In some cases, fruit and vegetables may have been incorporated into composite foods and thus may not be reported. The majority of consumption was associated with vegetables followed by fruit, fruit juice and pulses. There was no statistically significant difference in total fruit and vegetable consumption between males and females.

**Table 1:** Consumption of fruit and vegetables (F&V) by UK adults (g/d)* (Means, ranges and 95th percentiles (P95))

	Fruit (*n* 830)	Juice (*n* 412)	Pulses (*n* 419)	Vegetables (*n* 989)	Total F&V (*n* 1016)	Total F&V, men (*n* 447)	Total F&V, women (*n* 569)	BMI (*n* 1016) (kg/m^2^)
Mean	125	78	43	164	310	315	307	27
Range	0–1019	0–150†	0–80†	1–729	0–1400	0–1116	0–1400	17–51
P95	330	150†	80†	356	655	737	619	37

* F&V data derived from National Diet and Nutrition Survey^(^ ^14^ ^)^.

† Maximum consumption capped for health guidelines on F&V portion intake^(^ ^20^ ^)^.

For UK adults, 73 % were found to consume < 400 g/d of fruit and vegetables, and had an average consumption of 220 g/d. The remaining 27 % of adults who consumed >400 g/d had an average intake of 559 g/d. There were no apparent differences in BMI between the two groups (27 kg/m^2^), and no significant differences in fruit and vegetable consumption between males and females who did, or did not, meet the dietary guidelines (217 and 222 g/d, 559 and 540 g/d, average intake for males and females, respectively).

**Fig. 5:**
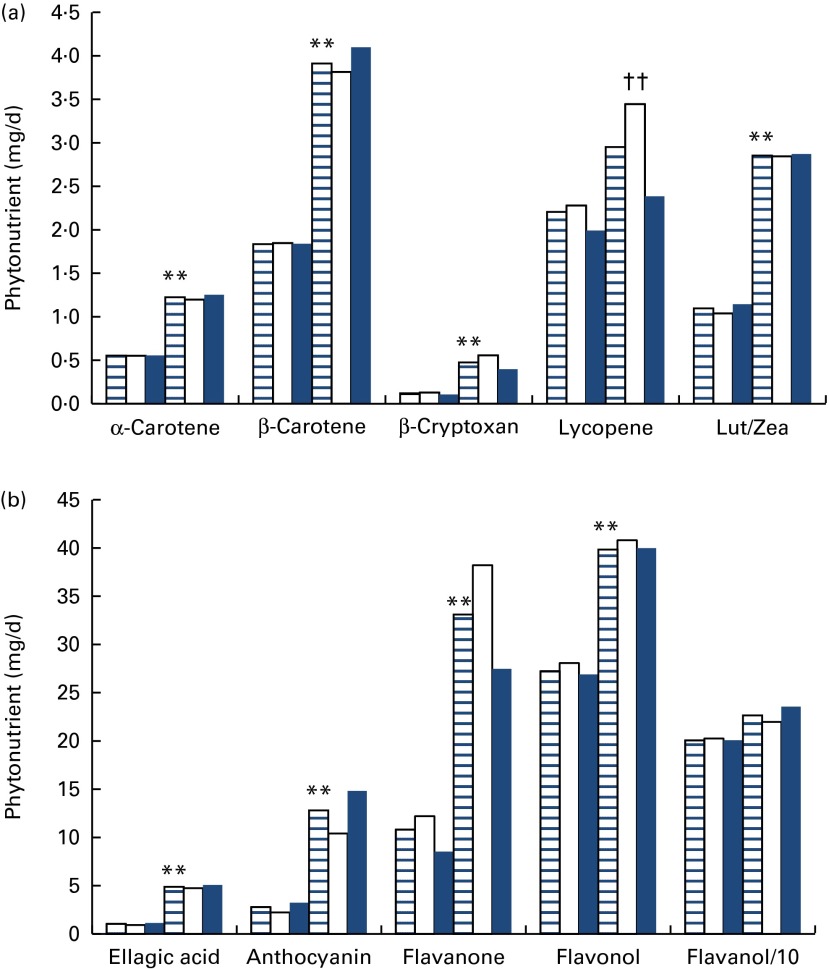
Average (a) carotenoid and (b) polyphenol intakes for consumers meeting or not meeting fruit and vegetable intake recommendations. 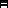, UK adults; 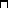, men; 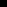, women. The first three bars for each phytonutrient represent < 400 g of average fruit and vegetable consumption; the second three bars represent >400 g of average fruit and vegetable consumption. Flavanols are scaled down by a factor of 10. ** Value was significantly different from that for UK adults, men and women consuming < 400 g of fruit and vegetables (*P*< 0·01). †† Value was significantly different from that for UK adults, and men consuming < 400 g of fruit and vegetables (*P*< 0·01). β-Cryptoxan is β-Cryptoxanthin, Lut/Zea is lutein and zeaxanthin. A colour version of this figure can be found online at http://www.journals.cambridge.org/bjn

Phytonutrient intakes were estimated for UK adults (Table 2). Men and women had similar levels of intake for all phytonutrients except lycopene (*P*< 0·01) and flavanones (*P*< 0·01), which were significantly lower in women (average, range and 95th percentile), and anthocyanidins (*P*< 0·03), which were significantly lower in men. Fig. 5 shows the difference in phytonutrient intakes between the individuals consuming more or less than 400 g/d of fruit and vegetables. Phytonutrient intake was significantly higher with greater fruit and vegetable consumption in every case except for flavanols, where no difference was observed, and lycopene which did not show a significant increase for women. There was also a difference in anthocyanin intake for men and women consuming higher levels of fruit and vegetables (*P*< 0·01). This was not observed with ellagic acid, despite the similar food sources of these two phytonutrients.

**Table 2:** Phytonutrient intakes from fruit and vegetables (F&V) in UK adults* (Means, ranges and 95th percentiles (P95))

	F&V (g/d)	α-Carotene	β-Carotene	β-Cryptoxanthin	Lycopene	Lutein/zeaxanthin	Anthocyanidin	Flavanone	Flavonol	Flavanol	Ellagic acid
UK adults											
Mean	310	0·73	2·4	0·2	2·4	1·6	5·5	17	31	208	2·1
Range	0–1400	0–5·95	0–16·8	0–6·0	0–32·2	0–11·3	0–279·6	0–270	0–135	0–1469	0–67·3
P95	655	2·42	6·8	0·9	7·8	5·1	29·1	69	68	572	15·7
UK males											
Mean	315	0·72	2·4	0·2	2·6	1·5	4·4	19	31	207	1·9
Range	0–1116	0–5·95	0–16·3	0–6·0	0–32·2	0–10·4	0–171·3	0–270	0–135	0–1469	0–67·3
P95	737	2·43	6·8	1·0	8·2	5·0	26·5	72	69	562	13·3
UK females											
Mean	307	0·74	2·4	0·2	2·1	1·6	6·3	14	30	210	2·2
Range	0–1400	0–5·70	0–16·8	0–2·0	0–16·8	0–11·3	0–279·6	0–105	0–107	0–1174	0–60·5
P95	619	2·41	7·1	0·8	6·8	5·1	29·1	55	68	576	16·4

* F&V consumption taken from the National Diet and Nutrition Survey data^(^ ^14^ ^)^. Phytonutrient intake in mg/d (range) is the product of individual F&V consumption and phytonutrient levels from online supplementary Table S1.

The effect of seasonality on phytonutrient intake was investigated for UK adults. A trend was observed for anthocyanins and ellagic acid, where intakes were highest in summer (see online supplementary Fig. S1).

## Uncertainty analysis

The WHO published guidelines for the characterisation and communication of uncertainty in exposure assessment that describe three main sources of uncertainty: scenario; model; parameters (WHO 2008)^(^ ^23^ ^)^. Specifically relevant to the assessment of phytonutrient intake are scenario uncertainties that may arise from the presence of phytonutrients in food additives and supplements that have not been taken into account and could have an impact on the intakes of certain sub-populations or individuals. Model uncertainties in this assessment are minor because relatively simple models were applied and the results were used to identify broad trends and not to characterise individual intakes.

Parameter uncertainties are more critical because they relate to the data available on phytonutrient levels in foods and the quantities of fruit and vegetables consumed. Where possible, phytonutrient levels were based on European data, but the results were subject to errors resulting from sampling, analysis and the presence of plant products in composite foods, affecting the raw data and potentially resulting in the phytonutrient contribution to the diet being misreported or unrecorded. The quality of food consumption data varied between surveys, even within survey types, and reflected uncertainties common to all types of nutritional analysis. FBS data are based on commodities in trade and so no distinction can be made between fruit and vegetables consumed raw and those processed into other products such as juices. The EFSA Comprehensive Database provides a greater level of detail; however, it was not possible to distinguish, for example, between green and black teas and so an arbitrary 50:50 divide was allocated. The UK NDNS data are based on individual weighed records. All food items that specifically mention a fruit and vegetable were included, but there were composite foods that comprised fruit and vegetables in part, which it was not possible to incorporate into the analysis. The relatively short duration of food consumption surveys will introduce uncertainty, in particular for infrequently consumed foods. It is likely that if the survey period was extended, then more consumers of a particular commodity would be identified and the amounts apparently consumed would decrease.

The overall effect of uncertainties in the assessments affects different methods in the tiered approach differently. More aggregated FBS data make it more likely that total fruit and vegetable consumption will have been overestimated because it is not possible to identify all varieties and form separately. Vegetables that would not normally be included in the WHO targets cannot be easily excluded and wastage due to preparation techniques may not be taken into account. The FBS data also cover entire populations so that it is not possible to consider adults separately resulting in a small underestimation. Estimates of phytonutrient intake are also probably reduced because particular fruit and vegetable varieties high in phytonutrients cannot be identified. The EFSA Comprehensive Database provides more information about foods ‘as eaten’ and so is likely to give a better estimate of both fruit and vegetable consumption and phytonutrient intakes. However, because the database contains data from national surveys that did not use the same survey methodologies, direct comparisons are less reliable. The UK NDNS data probably provide the most sound basis for assessment. However, the relatively short survey period and the potential omission of phytonutrients incorporated into composite foods may introduce uncertainties. The main sources of uncertainty can be summarised using the semi-quantitative method recommended by the EFSA Scientific Committee^(^ ^24^ ^)^ (Table 3).

**Table 3:** Simple semi-quantitative uncertainties associated with phytonutrient estimation

	Effect of uncertainty on the estimates of
Source of uncertainty	Fruit and vegetable consumption	Phytonutrient intake
Phytonutrient levels	NA	− /++
Food consumption data (FBS)	++	−
Food consumption data (EFSA Comprehensive)	− /+	− /+
Food consumption data (UK NDNS)	− /+	− /+

NA, not available; FBS, food balance sheets; EFSA, European Food Safety Authority; NDNS, National Diet and Nutrition Survey.

## Discussion

**Fig. 6:**
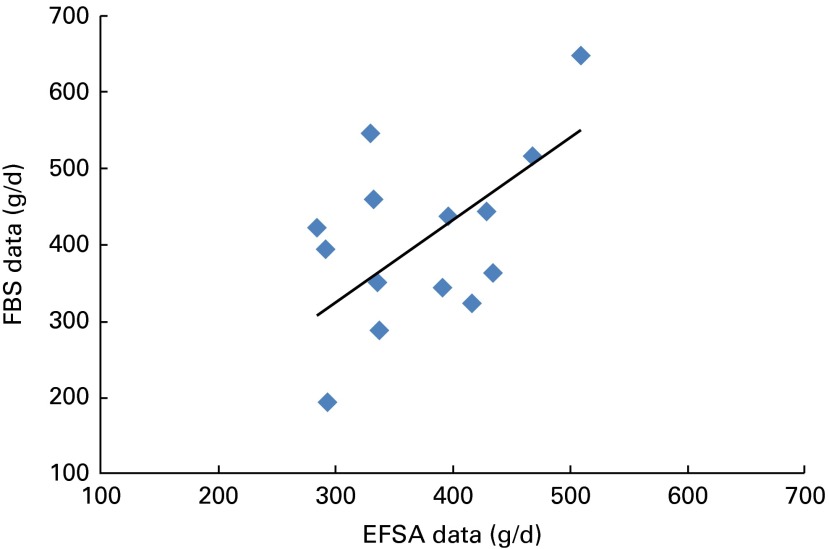
Correlation between European fruit and vegetable consumption assessed using the European Food Safety Authority (EFSA) Comprehensive Database and the WHO's food balance sheets (FBS = EFSA × 1·08, *R* ^2^ 0·234, *y*= 1·0809*x*). A colour version of this figure can be found online at http://www.journals.cambridge.org/bjn

Estimates of average long-term per capita consumption of fruit and vegetables in European countries based on the FAO's FBS data ranged from 170 to over 800 g/d. The range was wider than when the EFSA Comprehensive Food Consumption data were considered, indicating that the results may be related to food consumption survey methodology. Since the FBS data are based on annual trade statistics, they provide a more stable basis, but may not be sensitive to differences in home production or to losses related to food preparation. The EFSA data relate to food as consumed, but are frequently based on surveys of 2 or 3 d duration and so daily fluctuations in consumption patterns may influence the results. These differences mean that direct comparisons between countries are difficult to make. For example, while Sweden is among the higher fruit and vegetable-consuming countries in the FBS dataset, it appears as the lowest-consuming country in the EFSA database. This may relate more to survey methodology (e.g. recording foods as prepared dishes instead of raw agricultural commodities) than reflecting real differences. The weak correlation observed between the datasets (*r* ^2^ 0·234) reflects the differences in survey methodologies (Fig. 6). As a consequence, the results should be interpreted as broad trends rather than identifying detailed differences between countries. Under-reporting of foods is also known to cause misrepresentation of intake from dietary surveys, particularly energy intake; however, this is not necessarily the case with fruit and vegetable intake as some respondents may be more likely to overestimate their intake^(^ ^25^ ^)^.

Differences in survey methodology are accentuated when estimating intakes of phytonutrients because the FBS data are based on highly aggregated food groups that make it necessary to assume that all foods contain a given level of phytonutrients. This results in significant overestimates of true intakes, although inter-country comparisons may remain valid. The EFSA Comprehensive data are more disaggregated that the FBS data, and so it is possible to assign phytonutrient concentrations more precisely. This results in lower average estimates of intake of phytonutrients using these data. The UK NDNS data provide the most reliable basis for estimating intakes because the food descriptions are highly detailed so that phytonutrient levels can be precisely matched. It is then possible to estimate the intake of each individual in the survey to derive population statistics. According to the FBS data, total fruit and vegetable consumption in the UK was 436 g/d. The average consumption of UK adults using the EFSA Comprehensive data fell to 333 g/d, while the average consumption based on the NDNS data was 310 g/d (this agreement is to be expected because the data are derived from the same source).

The effects of different dietary surveys and data presentation have a more pronounced effect on the estimates of phytonutrient intake. Taking the UK again, intakes of anthocyanins were 75 mg/d based on the FBS data, 14 mg/d based on the European Union Comprehensive data and 5·5 mg/d based on the NDNS data. This indicates that absolute values for phytonutrient intake are difficult to predict from highly aggregated food consumption data and that only overall trends can be observed.

**Fig. 7:**
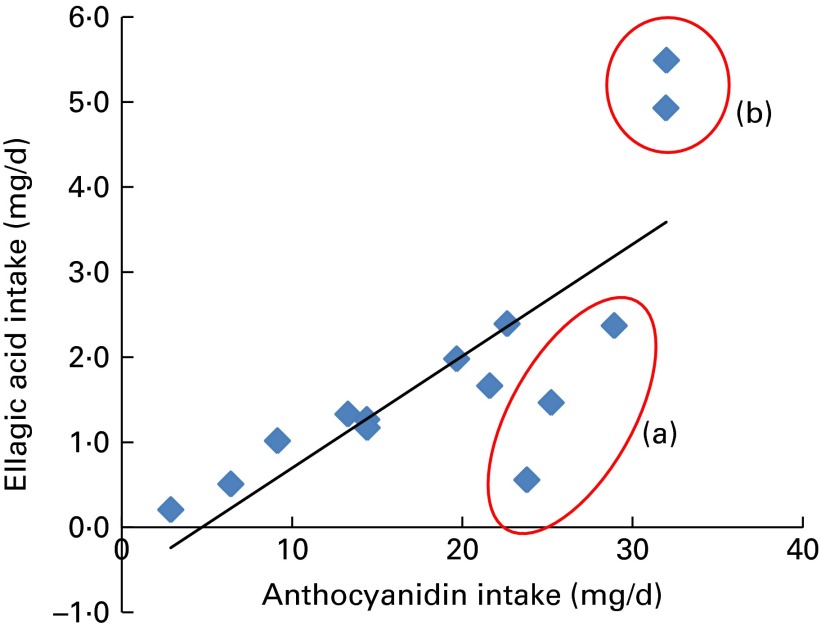
Correlation between the intakes of anthocyanins and ellagic acid using the European Food Safety Authority data^(^ ^19^ ^)^. Data in circles represent (a) high red wine or (b) high berry consuming countries. A colour version of this figure can be found online at http://www.journals.cambridge.org/bjn

The data presented in Tables 1 and 2 show that consumption of fruit and vegetables is a good predictor of phytonutrient intake, as would be expected. However, this is not always the case, and for some phytonutrients, the trend may be reversed. For example, the principal dietary source of lycopene is tomatoes, but processed foods containing tomatoes tend to have even higher lycopene levels than an equivalent amount of fresh tomatoes^(^ ^26^ ^)^. This indicates that to be a high consumer of lycopene, it is not necessary to be a high consumer of fresh tomatoes. Thus, the greater consumption in some countries of tomatoes in processed foods, such as sauces, may make a substantial contribution to lycopene intake unrelated to the intake of fruit and vegetables. This also helps to explain the lack of correlation of lycopene with fruit and vegetable intake (Fig. 4), and indicates why lycopene intake across the three different dietary survey methods did not alter significantly (3, 2·3 and 2·4 mg/d for FBS, EFSA and NDNS, respectively). Similarly, the consumption of certain berry fruits may form a higher proportion of total fruit and vegetable consumption in some northern European countries, leading to a higher intake of phytonutrients associated with these fruits (anthocyanins and ellagic acid). Fig. 7 shows the correlation between anthocyanins and ellagic acid from the EFSA data; the circles represent countries with dominance in high berry consumption (Finland and Latvia) or red wine consumption (Italy, Spain and France). Furthermore, the consumption of berry fruits may also be higher during the summer months when there is greater seasonal availability.

Murphy *et al.* ^(^ ^11^ ^)^ determined the intake of phytonutrients in high and low fruit and vegetable consumers in the US population. The results obtained from the NDNS data on the UK population are comparable to the US population for all the carotenoids, except lycopene which is three times higher in the USA compared with the UK for both the high and low fruit and vegetable consumers. The difference was suggested to be attributed to the higher intake of lycopene from raw tomatoes in the UK and from pasta sauces in the USA^(^ ^27^ ^)^. Among the polyphenols assessed, anthocyanins and ellagic acid were also found to be approximately three to five times higher in both high and low fruit and vegetable-consuming US adults. However, flavanones and flavonols were found to be higher in the UK population. This may be a result of higher citrus juice and tea consumption in the UK, respectively.

The flavanol intake found from the FBS and EFSA data (22 and 9 mg/d, respectively, for the UK) did not take into account tea consumption, which is a major source of non-fruit and vegetable-derived flavanols. The NDNS data for the UK did include tea consumption and thus demonstrated the significant impact of tea on this class of phytonutrient with 210 mg/d of these compounds consumed. The value from the NDNS data is similar to the data obtained for flavanol monomers from the European Prospective Investigation into Cancer and Nutrition (EPIC) UK cohorts^(^ ^28^ ^)^, which determined an intake of 149–215 mg/d. Mediterranean countries, which typically consume little tea, had a flavanol intake of 20–70 mg/d^(^ ^29^ ^)^. Likewise, tea is a major source of flavonols, and thus there is a significant increase in intake for the UK population based on the EFSA data (16 mg/d), in which tea was not taken into account, and the NDNS data (31 mg/d). Flavonol intake from the NDNS data was lower than that found in the EPIC UK cohorts^(^ ^30^ ^)^: 42–55 mg/d in the UK, 15–30 mg/d in Italy and 17–20 mg/d in Sweden.

The intake of ellagic acid derived from ellagitannins in the diet has until recently been more difficult to determine based on the limited compositional data available. Nuts are also a major source of ellagic acid, which were not determined in the present study, thus the level of 2 mg/d is an underestimation for UK intake. The range of ellagic acid is also highly skewed, as would be expected for a compound from a limited range of food sources, with the highest intake being thirty-two times the average amount consumed. Previous studies have suggested that ellagic acid intake in the UK may be about 5 mg/d^(^ ^31^ ^)^, but with some individuals, it may be >100 mg/d if pomegranate, berries or walnuts are regularly consumed^(^ ^32^ ^)^.

The difference in phytonutrient intakes between high and low fruit and vegetable consumers in the UK is marginal for flavanols (1·2 × ), flavonols (1·5 × ) and lycopene (1·2 × ), due to the relationship with tea and tomato products. The discrepancy in phytonutrient intakes between consumers with above and below the recommended fruit and vegetable intake levels is more significant for the other carotenoids (2·2–4 × ), flavanones (3 × ), anthocyanins and ellagic acid (5 × ). Thus, low fruit and vegetable consumers may be more likely to have low intakes of these potentially bioactive compounds.

In order for the phytonutrients to have systemic bioactivity, they need to be absorbed from the gastrointestinal tract. There is a large inter-individual variation in the absorption of both the polyphenols and the carotenoids, and thus the health benefit is not solely related to adequate intake. For example, for the flavanones, Brett *et al.* ^(^ ^33^ ^)^ found 0–57 % absorption and excretion of hesperetin from orange juice in 129 subjects. Combined with an average of three times difference in intake will result in a substantial difference in the potential benefits offered by these biologically active compounds.

### Conclusion

While the average consumer across Europe appears to consume nearly the recommended amount of 400 g/d of fruit and vegetables, there is a significant degree of variation. Some of this variation is between national diets, and the average consumption can vary by up to 4-fold between some countries in southern Europe and their northern and central European counterparts. The proportion of the population that appear to be meeting the nutritional guidelines can therefore vary from as few as one-third to as much as two-thirds. There is also considerable variation within populations so that some individuals appear to report very little or no consumption, whereas others appear to consume as much as three times or more than the amounts recommended by international authorities^(^ ^2^ ^)^.

To a large extent, phytonutrient intakes correspond to the consumption of fruit and vegetables and so higher consumers of fruit and vegetables are more likely to obtain the benefits of greater phytonutrient intakes. However, this is not always the case, and there are situations where processed foods may contain high levels of phytonutrients or particular national preferences may result in higher intakes. The seasonal availability of food is probably less important in recent years as food production becomes more global. However, there may still be significant effects, particularly for low-income groups who are unable to afford out-of-season fruit and vegetables.

Overall, intakes of phytonutrients are highly variable, suggesting that while some individuals are regularly consuming advised amounts^(^ ^2^ ^)^, there may be others who may not obtain all of the potential benefits associated with phytonutrients in the diet.

## Supplementary material

To view supplementary material for this article, please visit http://dx.doi.org/10.1017/S0007114514001950
